# Spontaneous Color Preferences and Associative Learning in *Protaetia brevitarsis* (Coleoptera: Scarabaeidae)

**DOI:** 10.3390/insects15100780

**Published:** 2024-10-08

**Authors:** Hui Wu, Zhuangzhi Cui, Xiaoqing Huang, Khalid Hussain Dhiloo, Fanfang Kong, Zhongyue Wang, Yongqiang Liu

**Affiliations:** 1 State Key Laboratory for Biology of Plant Diseases and Insect Pests, Institute of Plant Protection, Chinese Academy of Agricultural Sciences, Beijing 100193, China; 2 Department of Entomology, Faculty of Crop Protection, Sindh Agriculture University, Tandojam 70060, Pakistan

**Keywords:** *Protaetia brevitarsis*, grape, color preference, spectral characteristics, color learning

## Abstract

**Simple Summary:**

Adult *Protaetia brevitarsis* (Coleoptera: Scarabaeidae, Lewis) is an omnivorous beetle that damages primarily crops and fruits, especially grapes. The objective of our study was to gain insight into vision and olfaction in *P. brevitarsis* adults. The results indicated that the adults innately preferred wavelengths in the red spectrum, particularly 730 nm. It is the first report that *P. brevitarsis* adults had an associative learning ability, which meant they could associate the feeding environment with a food reward. This is of great importance in revealing the feeding preferences of adults and their persistent damage in vineyards. And we also found that vision was not the only factor in foraging decisions; olfactory cues also influenced their decision making. These results contribute to our understanding of the combined role of visual and olfactory cues for host localization and provide a basis for designing effective traps for *P. brevitarsis* adults.

**Abstract:**

Color vision, which varies among species, plays an important role in foraging, mating, and habitat selection among insects. *Protaetia brevitarsis* (Coleoptera: Scarabaeidae, Lewis) is an omnivorous beetle that damages both crops and fruit. Here, to understand the effect of vision and olfaction in host selection, experiments were conducted on the spectral wavelength preference, color preference, and associative learning ability of adult *P. brevitarsis* using LED lights and grapes. In our experiments, adults showed the strongest spontaneous preference toward the red spectrum, particularly 730 nm. Non-preferred lights were used to train adults with a food reward (grapes). Green-trained adults had an increasing tendency to prefer green light, and blue-trained adults had a clear preference for blue light. Furthermore, adults significantly preferred red grapes in the absence of olfactory cues, but their selectivity for grapes differed in the presence of olfactory cues, indicating that vision was not the only factor in foraging decisions, but that olfactory cues also influenced their decision making. The results lay the groundwork for revealing their host localization mechanism and provide promising avenues for biological control in the field.

## 1. Introduction

Vision is common in insects. For example, polarized light, though imperceptible to the human eye, can impact the host localization and migration of insects [1,2]; the physical features of host plants can impact the feeding, oviposition, and positioning behaviors of certain insects [3,4]; leaves, buds, bracts, flowers, and fruits of plants vary in their spectral reflectance characteristics, which insects can use to distinguish plants [5,6]. These findings also suggest that a preference for a particular color is not only a visual cue for insects but also affects activities such as communication, mating, and reproduction [7]. Spectroscopic features always play an important role in insect vision, but they are not the sole source of visual information for insects [8]. Compound eyes, composed of tens of thousands of ommatidia, are the primary visual organ of insects; in these ommatidia, different spectral photoreceptors occupy the visual axis and field, allowing the perception of color at each spatial pixel [9,10]. Insects utilize visual stimuli to locate host plants at close range [11]. Indoor simulations have revealed that reflected light from plant hosts can generate attraction to insects [12]. In beetles, the opsin gene subfamily that confers sensitivity to “blue” wavelengths has been lost [13], but physiological evidence suggests that many beetles have blue-sensitive photoreceptors. Recent gene family studies suggest that blue sensitivity has been regained in these cases through the duplication and subfunctionalization of the ultraviolet (UV) and long-wave length (LW) sensitive opsin genes [14]. In the flower-visiting beetle *Heterochelus* sp. (Scarabaeidae), for instance, blue opsin has also been lost, but LW opsin is duplicated (one UV duplication event and two LW duplication events) [13]. Furthermore, olfaction is common in insects for searching for host plants as well, but in some insects, olfactory cues may be less important than visual cues, or they work synergistically [15,16]. For example, beetles (*Altica engstroemi*) are significantly drawn to visual stimuli but do not react to olfactory stimuli [17]. Adult monophagous leaf beetles (*Ambrostoma quadriimpressum*) fail to discern hosts from a distance, but they can be attracted by standing visual targets when searching for potential hosts [18]. These previous studies support the hypothesis that beetles may navigate and land on objects using visual cues and subsequently use other cues to distinguish appropriate hosts [19].

*Protaetia brevitarsis* (Coleoptera: Scarabaeidae, Lewis) adults are omnivorous beetles that damage primarily crops and fruits including maize, sunflower, cotton, grape, peach, and the tender shoots of other plants, and their sugar tropism makes them a serious threat to grapes [20,21]. The adults typically cause two types of damage to mature grapes. (1) The adults are voracious eaters and feed on grapes until only the skin remains, and (2) wounds from feeding can then become infected by pathogens [22]. Given that adults are present when the fruit ripens and chemical control poses concerns for food safety, biological control is predominantly used to control this pest. Interestingly, trapping results using different colored traps in the field and color preference tests using cardboard both indicated that the adults had a strong preference for red [23]. It is not clear how the adults identify and locate hosts for feeding, and we are also confused about how visual and olfactory cues influence their positioning on grapes. What caused their preference for red cardboard, is it because of their innate preference? Or is it because adults can learn to associate red with the ripe grapes they eat?

Extensive research has been conducted to determine the spontaneous color preferences of insects by utilizing artificial bloom, colored cardboard, and LED light to gain a deeper understanding of how visual cues affect insect behavior. In the current research, LED lights as a vital tool were widely used to carry out our experiment. Researchers using LED light have found that harlequin bugs (*Murgantia histrionica*) are most attracted to green light at 510 nm [23], and ants (*Camponotus blandus*) have a spontaneous preference for ultraviolet light at 365 nm [24]. Nevertheless, insects are complex and usually vary considerably in color preference across species and sex [25,26,27,28]. Research on sexual differences in spectral sensitivity has already provided noteworthy evidence at the molecular level in butterflies [29]. Thus, we also conducted experiments on male and female adults separately. Learning behaviors, in contrast to spontaneous responses including associative learning and aversive learning, given the complexity and capriciousness of the natural environment, are thought to provide insects with behavioral plasticity to adapt to variable surroundings [30,31,32]. In general, associative color learning has been demonstrated in flower-visiting insects including bees, butterflies, and moths, but rarely in the Scarabaeidae family [33,34,35], especially in *P. brevitarsis* adults, which have never been studied.

To clarify the influence of color and olfactory cues on adult host selection, here we tested adult preferences for monochromatic lights and preferences for four grape varieties that differ notably in appearance and taste. In addition, the adults were also trained with non-preferred lights to investigate whether adults have an associative color-learning ability. The results will provide a theoretical basis for developing strategies to capture and eliminate *P*. *brevitarsis* adults.

## 2. Materials and Methods

### 2.1. Insects

Newly emerged adults with a uniform body size were purchased from Shijialing Biodiverse Farm (Shandong, China). The males and females were raised in different reptile enclosures with a controlled temperature (26 ± 1 °C), relative humidity (75 ± 5%), photoperiod L:D (14 h:10 h) in the laboratory. When adults were not being tested, they were provided grapes and peaches from 10:00 am to 12:00 pm and 16:00 pm to 18:00 pm, and feeding behavior was more active during these two periods. When we conducted preference experiments, they required varying degrees of starvation to ensure that the adults were eager to choose food or light stimuli. In each experiment, 100 male and 100 female adults were separately tested with 3 replicates.

### 2.2. Stimuli

Two types of stimuli were set, including visual stimuli and olfactory stimuli. Visual stimuli, fruit color of varying grape species, and monochromatic light of varying wavelengths were set to initiate the color preference experiments. Grape scents were used as the olfactory stimuli. For the grape color stimuli, ripe grapes of four species with different colors were used directly. For light stimuli, 10-watt LED panels (Anhui Aihaidi Lighting Technology Company, Anhui, China) and a mobile application called LED Magic Light 2.3.0 (Publisher: Mingzhang Zhou) were both needed. They have built-in Bluetooth to connect to each other. After connecting, the LED Magic Light can turn on and off each LED panel separately; also, it can separately adjust RGB values to generate different light colors on each panel (RGB color is called additive color; R-red, G-green, and B-blue are used together to set the LED lights to generate different colors). The conversion function of Bruton (http://www.philiplaven.com/p19.html; accessed on 15 June 2023) was used to convert the selected wavelengths to RGB values. The chosen wavelengths and their corresponding RGB values are listed in Table 1 and Table 2.

### 2.3. Experimental Arena

**Figure 1:**
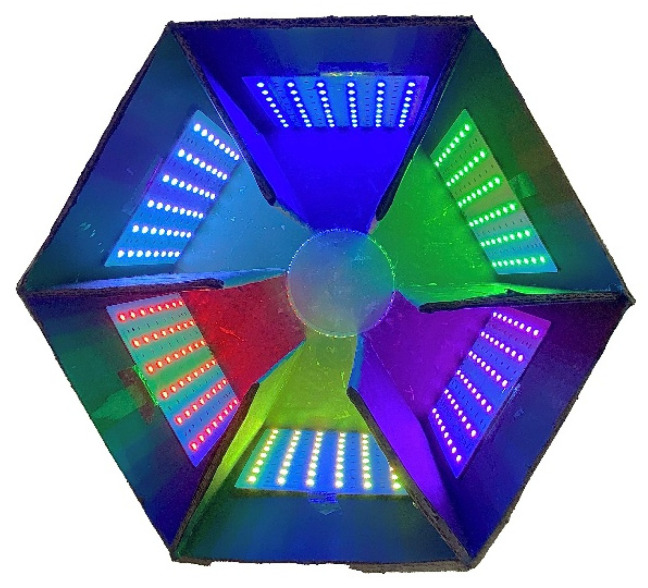
Hexagonal maze to test *Protaetia brevitarsis* adults for preferred wavelengths generated by LED panels. The transparent glass bottle was inverted in the center, allowing adults to move freely and fully observe the color of each chamber.

**Figure 2:**
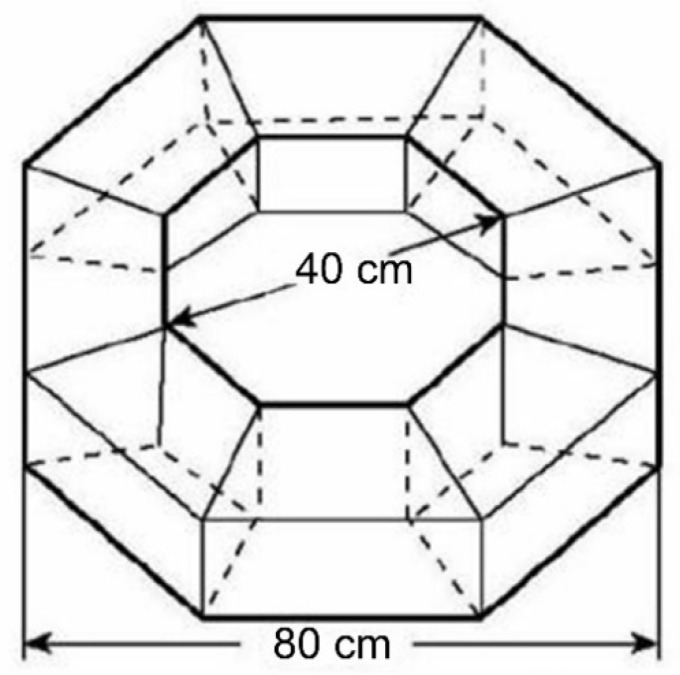
A schematic of the octagonal maze for testing *Protaetia brevitarsis* adults for grape preference. The maze was designed by Tang et al. [36].

We used black cardboard, assembled into a hexagonal maze (base edge 20 cm, face height 23 cm, partition length 13 cm, partition height 23 cm) (Figure 1) and an octagonal maze (Figure 2) as the experimental arena, as described by Tang et al. [36] (Figure 2). LED panels (15 cm × 15 cm) were placed in each arena room; LED lights with adjusted RGB values and specific wavelengths were turned on when we conducted the color preference experiments. Also, light illumination was measured using a Sigma AS803 digital illuminometer (Sigma Technology, Shenzhen, China). Our experiments were conducted in a darkroom. The only stimuli were the LED light, grape color, and grape scent.

### 2.4. Test of Visual Preference for Specific Wavelengths

Initially, 6 wavelengths of 6 colors (purple, blue, green, yellow, red, and white (control) (Table 1) were tested, with each light being randomly placed in the hexagonal maze. For each test, 10 adults were placed simultaneously in the transparent glass bottle in the hexagonal maze. The adults were usually crawling around the bottle at this time, so we waited for 3 min to ensure that they had a clear view of 6 light stimuli, then the adults were released from the bottle to move freely. And they usually crawled into the arena rooms or onto the light panels or stayed in the center without moving. If the adults stayed in an arena room for more than 1 min, then the light stimulus was deemed as preferred by the adults. The observation was ended if the adults had not made any choice for over 3 min. Additionally, 6 light stimuli were repositioned after adults chose their preferred color. Then, the hexagonal maze was wiped with alcohol and air-dried to eliminate any effects of orientation and olfactory cues on the measurement results of the next group.

After that, 6 types of red stimuli in the red spectrum (Table 2) were also tested in the same way.

### 2.5. Test of Associative Color Learning

Two wavelengths, 520 nm (green) and 440 nm (blue), were chosen for the associative color-learning test because adults show no innate preference for either wavelength. The test consists of two parts: first, training the adults to learn color, and then, the adults choosing their preferred color. For the training part, in the hexagonal maze, all light panels were set to a non-preferred color (green or blue). A transparent glass dish containing fresh grapes was placed in the center of the hexagonal maze, and each time 10 adults starved for 48 h were placed in the dish, individuals returned to the enclosure after feeding for 1 min. After the adults were starved for another hour, the above training procedure was repeated. One hour after the second training, the selection part was conducted. Given that 730 nm is the most preferred wavelength, 650 nm was changed to 730 nm to investigate if learned behavior could cover their innate preference. For the choosing part, the light stimuli were adjusted according to Table 3. The color preference of the trained adults was then tested in the hexagonal maze using the method described in Section 2.4 for the visual preference test for specific spectra.

### 2.6. Selection Response to Visual Cues

Adult visual preference was tested using fruit color as the sole visual stimulus. Ripe grapes of Shine-Muscat, Red Globe, Ruby Seedless, and Melissa, 50 g, were transferred to four transparent flasks, and all flasks were sealed with transparent foil to prevent odor contamination. Each flask was placed in the room of the octagon maze (Figure 2), and there was a gap room between every two flasks to ensure consistent spacing. The light panels were adjusted to white (R: 255, G: 255, B: 255) to display the full fruit colors. Each time, 10 adults were placed in the center of the octagon maze to test their color preference, using the same method as described in Section 2.4 for the visual preference test for specific spectra. The flasks were also repositioned after each of the 10 adults selected their preferred color.

### 2.7. Selection Response to Odors Combined with Visual Cues

The method was exactly the same as the selection response to visual cues, except that the flasks were left unsealed to release their odors.

### 2.8. Statistical Analysis

SPSS 13.0 (SPSS, Chicago, IL, USA) was used for all analyses. All proportion data were arcsine square-root-transformed before analysis [37]. The mean (±SE) proportion of adults attracted by specific spectra and mean (±SE) proportion of adults attracted by grapes were analyzed separately using a one-way ANOVA. When the ANOVA indicated a significant difference, Tukey’s honestly significant difference (HSD) test was used to separate the means.

## 3. Results

### 3.1. Visual Preferences of P. brevitarsis for Specific Spectra

**Figure 3:**
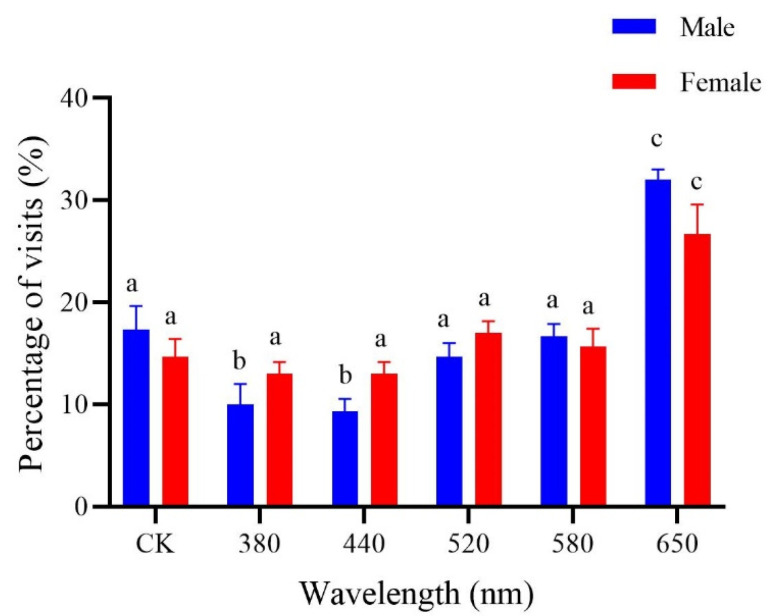
Percentage of visits of *Protaetia brevitarsis* adults to 6 light stimuli in diverse spectra. Wavelength colors: 380 nm, purple; 440 nm, blue; 520 nm, green; 580 nm, yellow; 650 nm, red; CK, white. Lowercase letters indicate significant differences among means at *p* < 0.05; the vertical line at the top of each histobar indicates standard error.

The male selection rate for 650 nm was the highest and differed significantly from the rates for the rest of the colors (*F*_5,12_ = 19.96, *p* < 0.001), with the lowest rates for 380 nm and 440 nm, but the selection rates did not differ significantly between 520 nm, 580 nm, and CK (Figure 3). Female adults also had the highest selection rate for 650 nm, which differed significantly from the rates for the other monochromatic stimuli (*F*_5,12_ = 8.17, *p* < 0.01); and the female selection rate for the remaining wavelengths did not differ significantly.

**Figure 4:**
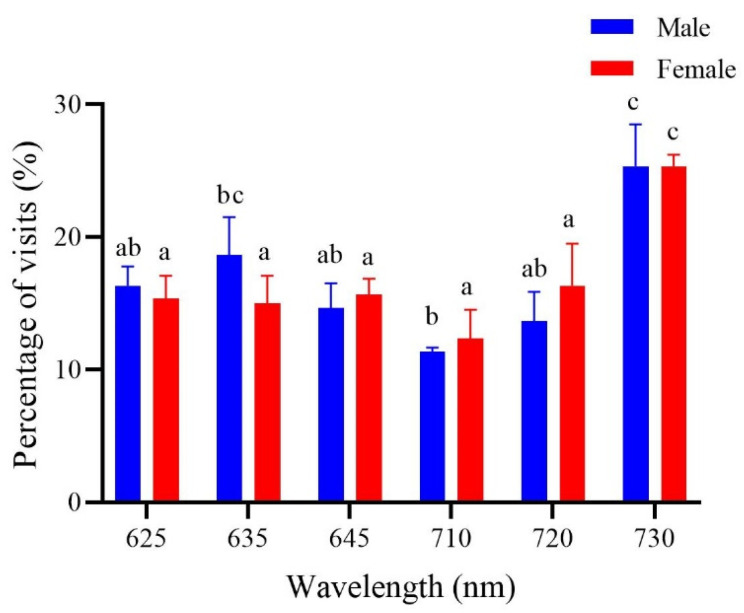
Percentage of visits of *Protaetia brevitarsis* adults to 6 light stimuli in the red spectrum. Lowercase letters indicate statistically significant differences existing among the treatments at the 0.05 level. Lowercase letters indicate significant differences among means at *p* < 0.05; the vertical line at the top of each histobar indicates standard error.

In the color preference test for the 6 types of red stimuli, the female preference for 730 nm was significantly higher than for the other 5 stimuli; but the males significantly preferred both 730 nm and 635 nm (Figure 4).

### 3.2. Associative Color Learning

**Figure 5:**
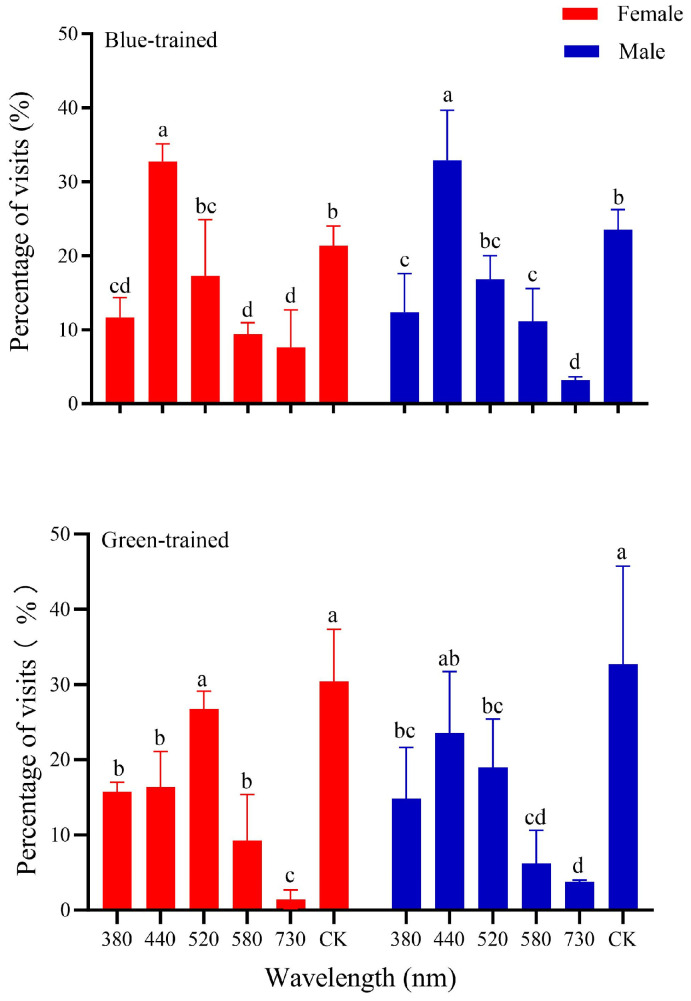
Percentage of visits of color-trained *Protaetia brevitarsis* adults to 6 wavelengths. Wavelengths and colors: 380 nm, purple; 440 nm, blue; 520 nm, green; 580 nm, yellow; 650 nm, red; control CK, white. Lowercase letters indicate significant differences among means at *p* < 0.05; the vertical line at the top of each histobar indicates standard error.

For the training part, adults kept staying in the transparent glass dish and eating grapes. For the selection part, there existed a difference between the green-trained and blue-trained adults. The green-trained adults showed that females significantly preferred 520 nm (green) and CK (white) (*F*_5,12_ = 18.03, *p* < 0.001); males preferred 520 nm (green), 440 nm (blue), and CK (white). And their innate preference for red light was significantly weaker after training (Figure 5). Blue-trained adults, both female and male, significantly more selected 440 nm (blue) (females: *F*_5,12_ = 14.99, *p* < 0.001; males: *F*_5,12_ = 17.73, *p* < 0.001). Blue light training could also override their innate preference for red light (Figure 5). Given that the color-trained adults had a stronger tendency toward training colors and a weaker tendency toward spontaneous preferred color, it is concluded that the trained adults associated color with food; in other words, they learned the color.

### 3.3. The Selection Preference for Grapes in the Absence of Odors

**Figure 6:**
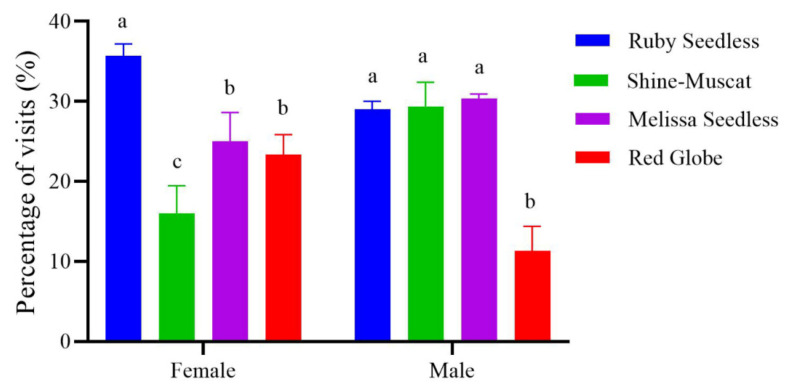
The percentage of visits of *Protaetia brevitarsis* adults to grapes of four varieties when the odors were blocked. The lowercase letters indicate significant differences among means at *p* < 0.05; the vertical line at the top of each histobar indicates standard error.

The female and male adults had a significantly higher selection rate for Ruby Seedless, followed by Red Globe; the rates for Shine-Muscat and Melissa Seedless were similarly lower. Since Ruby Seedless and Red Globe are grapes with red fruits, and Shine-Muscat and Melissa Seedless are grapes with green fruits, it is suggested that adults have a stronger preference for red species than green species (Figure 6).

### 3.4. The Selection Preference for Grapes in the Presence of Odors

**Figure 7:**
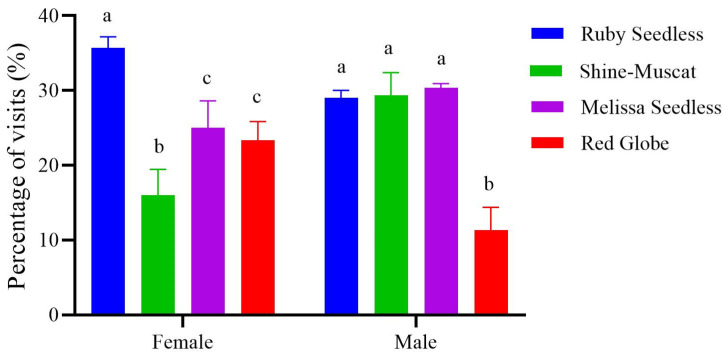
The selectivity of *Protaetia brevitarsis* adults to grape varieties when odors were present with visual cues. The lowercase letters indicate significant differences among means at *p* < 0.05; the vertical line at the top of each histobar indicates the standard error.

When both visual and olfactory stimuli were present, male and female adults differed in their preferences for the four varieties of grapes (Figure 7). Males did not differ significantly in their selection rate for Shine-Muscat, Ruby Seedless, and Melissa Seedless, but the male selection rate for Red Globe was significantly lower than for the other three species (*F*_3,8_ = 43.61, *p* < 0.001). However, the female selection rate for Ruby Seedless was significantly higher than for the other three varieties, and the selection rate for Sunshine Muscat was the lowest (*F*_3,8_ = 20.51, *p* < 0.001).

## 4. Discussion

The present study reported that *P. brevitarsis* adults, both male and female, spontaneously preferred wavelengths in the red spectrum, particularly 730 nm. The adults showed an associative color-learning ability. They could associate the color of the feeding environment with food feedback, and the female learning ability was superior to that of the male in our experiment. Further, we demonstrated that color vision was not the only factor in adult foraging decisions; odor cues also influenced their decision making, and this influence differed between males and females.

In the preference test for specific wavelengths, it was discovered that red light was preferred by adults, which is consistent with field experiments by Cai et al. [21], who trapped significantly more adults with red cardboard than with other cardboards. Our experiment first reported that *P. brevitarsis* adults spontaneously prefer 730 nm over the other wavelengths tested. Currently, visual research on beetles is relatively deficient, but related studies in the Scarabaeidae family indicate that they have a preference for certain wavelengths or colors. However, preference differences are common among species. For example, *Holotrichia parallela* and *Anomala corpulenta* adults are strongly attracted to light at 385 nm in the field [38]; yellow and white oil-painted traps captured more *Hoplia spectabilis* adults than the red-painted trap [39]. This interspecific difference is multifaceted due to inherent factors such as photoreceptor or visual opsins, which can result in responses to specific spectra of photon wavelengths—as is the case with *Pygopleurus israelitus*, a beetle with three types of photoreceptors, which are maximally sensitive in the UV, green, and red areas of the spectrum [40]—or due to environmental differences, meaning that insects with learning abilities associate their foraging environment with food feedback [41,42].

Male and female adults of *P. brevitarsis* were trained by two non-preferred lights; the adults were able to feed on grapes only when the training lights were on. Blue-trained male and female adults both had noticeably stronger preferences for blue light compared to other lights, which means they can effectively associate blue light with food rewards. This associative learning ability is commonly demonstrated in bees and butterflies but barely in beetles [35,41]. Yet, the preferences of the green-trained adults differed between the females and males. The females preferred white more than green, and the males preferred white and blue more than green. The results for green-trained adults are consistent with the findings for monarch butterflies (*Danaus plexippus*) by Blackiston et al. [33], who named this phenomenon “confusion”, which was measured as the extent to which butterflies visited “non-trained” colors. However, our data indicate that the adults can learn to associate green and blue light with food rewards and even override their innate preference for red light. Here, we also find the “confusion” phenomenon in *P. brevitarsis* adults. But additional studies are required to determine the specific causes of this “confusion”.

In the grape preference test, adult *P. brevitarsis* were influenced not only by visual cues but also by olfactory cues. Without olfactory cues, adults preferred red grapes to green grapes, specifically manifested as males and females preferring, in order, Ruby Seedless (red fruits), Red Globe (red fruits), Sunshine-Muscat (green fruits), and Melissa Seedless (green fruits). The selection rates for the four varieties differed significantly. This is consistent with our finding that adults prefer red light. But when visual and olfactory cues were both present, significantly more females were attracted to Ruby Seedless, and significantly more males were attracted to Ruby Seedless, followed by Sunshine-Muscat and Melissa Seedless. Apparently, adults changed their variety preferences in the presence of olfactory cues, suggesting that vision is not the only factor in foraging decisions. Olfactory cues also influence their decision making. Our results are similar to experiments with *M. mineus* in which females show no preference when color stimuli are presented without odor but show a clear color preference when both food odor and color are present [33]. In addition, *P. brevitarsis* males and females responded differently to the same stimulus as males and females of the melon fruit fly do (*Bactrocera cucurbitae*). In the absence of olfactory cues, female fruit flies significantly preferred 540 nm, in contrast to the more generalized preference [43]. Differences in spectral sensitivity among species can be caused by differences in photoreceptor cells, visual pigments, and opsins, or by different nutritional needs [9]. Such differences may be due to the diversity of visual pigments that absorb different photons and convert the photons into electrical impulses in insects [44].

Our findings may provide new avenues for studying how *P. brevitarsis* adults identify their hosts. Simultaneously, it is established that vision and olfaction work synergistically; therefore, light traps and trap boards can be set in the field based on their visual preferences. And further investigation into the olfactory preferences of adults is required. Once this has been conducted, attractants can be designed and combined with insect traps to prevent and control *P. brevitarsis* adults specifically.

## Figures and Tables

**Table 1:** Specific wavelengths and corresponding RGB values for color preference test.

Wavelength (nm)	R	G	B	Illumination (lx)
380	97	0	97	1400
440	0	0	255	2360
520	54	255	0	2100
580	255	255	0	2430
650	255	0	0	600
CK (white)	255	255	255	4500

**Table 2:** Red spectrum and corresponding RGB values for color preference test.

Wavelength (nm)	R	G	B	Illumination (lx)
625	255	78	0	1230
635	255	39	0	900
645–700	255	0	0	600
710	233	0	0	530
720	210	0	0	500
730	188	0	0	460

**Table 3:** Specific wavelengths and corresponding RGB values for associative color learning.

Wavelength (nm)	R	G	B	Illumination (lx)
380	97	0	97	1400
440	0	0	255	2360
520	54	255	0	2100
580	255	255	0	2430
730	188	0	0	460
CK (white)	255	255	255	4500

## Data Availability

All data generated or analyzed during this study are included in this published article.
